# Supplementary material for: Classification and Functional Analysis between Cancer and Normal Tissues Using Explainable Pathway Deep Learning through RNA-Sequencing Gene Expression
Source: Int J Mol Sci. 2021 Oct 26;22(21):11531. doi: 10.3390/ijms222111531 (PMC8584109; doi:10.3390/ijms222111531)
Supplement: Supplementary file 1 [file ijms-22-11531-s001.zip › TableS1.pdf]

Table S1 Pathway contribution gene index

Gene symbol	Pathway contribution gene index
PLA2G10	1.149
SHC1	0.904
CDK1	0.846
CD4	0.779
APOA1	0.721
HIST1H3A	0.663
CDC16	0.647
LHB	0.622
PLA2G2E	0.616
ORC6	0.610
HIST1H4K	0.557
GNG11	0.542
TPM1	0.537
CHRNB4	0.525
CALM1	0.520
CAN	0.519
HLA	0.508
HIST1H4D	0.495
PLA2G4D	0.489
MYH6	0.487
MCM6	0.479

TPM3	0.479
FGF3	0.477
CDC45	0.477
ABCG1	0.468
PPP2R1B	0.456
CTNNB1	0.453
HYAL1	0.452
PLA2G12A	0.450
NRP2	0.450
HIST1H4I	0.447
LCAT	0.443
RAC1	0.435
CD3E	0.433
MBL2	0.430
SI	0.428
GNGT2	0.427
RAP1A	0.412
CYP21A2	0.410
PRKCI	0.410
CDC25C	0.406
GNAS	0.400
STAT1	0.397
HIST2H2AA4	0.397
CHRNA3	0.396

CDH3	0.394
HAAO	0.391
OGN	0.390
PLA2G2A	0.388
PSMD13	0.383
PSMF1	0.382
PAK1	0.381
DHFR	0.379
PPP2R5D	0.376
CCNE1	0.373
CRP	0.371
HTR7	0.368
