# Supplementary material for: Classification and Functional Analysis between Cancer and Normal Tissues Using Explainable Pathway Deep Learning through RNA-Sequencing Gene Expression
Source: Int J Mol Sci. 2021 Oct 26;22(21):11531. doi: 10.3390/ijms222111531 (PMC8584109; doi:10.3390/ijms222111531)
Supplement: Supplementary file 1 [file ijms-22-11531-s001.zip › TableS2.pdf]

Table S2 Pathway contribution gene index Top 1% Enrichment test (FDR < 1E-03)

Function	FDR
G1/S transition of mitotic cell cycle	1.03E-12
DNA replication	2.52E-12
DNA-dependent DNA replication	1.25E-09
DNA strand elongation involved in DNA replication	2.89E-09
DNA strand elongation	4.15E-09
MCM complex	4.15E-09
phosphatidylinositol acyl-chain remodeling	6.07E-08
phosphatidylserine acyl-chain remodeling	7.28E-08
phosphatidylglycerol acyl-chain remodeling	7.28E-08
cellular amine metabolic process	1.12E-07
amine metabolic process	1.35E-07
phosphatidylcholine metabolic process	1.35E-07
phosphatidylethanolamine acyl-chain remodeling	4.04E-07
phosphatidylserine metabolic process	4.98E-07
ethanolamine-containing compound metabolic process	5.66E-07
phosphatidylcholine acyl-chain remodeling	5.72E-07
cellular biogenic amine metabolic process	6.11E-07
alditol phosphate metabolic process	8.44E-07
phosphatidic acid biosynthetic process	1.21E-06
phosphatidic acid metabolic process	1.21E-06
Sarcomere	1.50E-06

phosphatidylglycerol metabolic process	2.54E-06
Myofibril	4.89E-06
alcohol metabolic process	4.89E-06
contractile fiber part	4.89E-06
contractile fiber	9.82E-06
cellular modified amino acid metabolic process	6.66E-05
striated muscle thin filament	1.02E-04
Myofilament	2.49E-04
muscle filament sliding	2.53E-04
actin-myosin filament sliding	2.53E-04
glycerophospholipid biosynthetic process	3.64E-04
DNA replication initiation	4.61E-04
phospholipid biosynthetic process	5.30E-04
actin-mediated cell contraction	6.48E-04
regulation of transcription involved in G1/S transition of mitotic cell cycle	8.81E-04
plasma lipoprotein particle remodeling	8.81E-04
protein-lipid complex remodeling	8.81E-04
macromolecular complex remodeling	8.81E-04
glycerophospholipid metabolic process	9.03E-04
glycerolipid biosynthetic process	9.77E-04
