# Supplementary material for: Classification and Functional Analysis between Cancer and Normal Tissues Using Explainable Pathway Deep Learning through RNA-Sequencing Gene Expression
Source: Int J Mol Sci. 2021 Oct 26;22(21):11531. doi: 10.3390/ijms222111531 (PMC8584109; doi:10.3390/ijms222111531)
Supplement: Supplementary file 1 [file ijms-22-11531-s001.zip › TableS3.pdf]

Table S3 Cancer-related c2 reactome 42 pathways and one sample z-test FDR q-value of pathways (FDR q-value < 0.05)

Pathway	FDR q-value
REACTOME_GPCR_DOWNSTREAM_SIGNALING	0
REACTOME_GPCR_LIGAND_BINDING	4.54E-100
REACTOME_NEURONAL_SYSTEM	9.86E-73
REACTOME_SLC_MEDIATED_TRANSMEMBRANE_TRANSPORT	1.10E-64
REACTOME_SIGNALING_BY_EGFR_IN_CANCER	4.13E-55
REACTOME_AXON_GUIDANCE	6.81E-53
REACTOME_PEPTIDE_LIGAND_BINDING_RECEPTORS	1.31E-29
REACTOME_DIABETES_PATHWAYS	9.06E-20
REACTOME_GASTRIN_CREB_SIGNALLING_PATHWAY_VIA_PKC_AND_MAPK	4.20E-17
REACTOME_SIGNALING_BY_FGFR_MUTANTS	9.46E-17
REACTOME_COLLAGEN_FORMATION	3.74E-16
REACTOME_FATTY_ACYL_COA_BIOSYNTHESIS	3.42E-15
REACTOME_CIRCADIAN_REPRESSION_OF_EXPRESSION_BY_REV_ERBA	8.17E-12
REACTOME_NFKB_IS_ACTIVATED_AND_SIGNALS_SURVIVAL	1.73E-11
REACTOME_ACETYLCHOLINE_BINDING_AND_DOWNSTREAM_EVENTS	6.24E-11

REACTOME_G_ALPHA_Q_SIGNALLING_EVENTS	2.89E-09
REACTOME_METABOLISM_OF_CARBOHYDRATES	6.25E-09
REACTOME_STRIATED_MUSCLE_CONTRACTION	1.78E-08
REACTOME_PI_3K_CASCADE	1.02E-07
REACTOME_NUCLEAR_SIGNALING_BY_ERBB4	4.35E-06
REACTOME_DEGRADATION_OF_THE_EXTRACELLULAR_MATRIX	4.63E-06
REACTOME_ACYL_CHAIN_REMODELLING_OF_PE	2.90E-05
REACTOME_VEGF_LIGAND_RECEPTOR_INTERACTIONS	6.52E-05
REACTOME_PRESYNAPTIC_NICOTINIC_ACETYLCHOLINE_RECEPTORS	6.64E-05
REACTOME_SIGNALING_BY_NOTCH3	1.42.E-04
REACTOME_MUSCLE_CONTRACTION	1.54.E-04
REACTOME_ACYL_CHAIN_REMODELLING_OF_PG	3.08.E-04
REACTOME_BRANCHED_CHAIN_AMINO_ACID_CATABOLISM	3.75.E-04
REACTOME_ENDOGENOUS_STEROLS	3.84.E-04
REACTOME_PURINE_CATABOLISM	9.34.E-04
REACTOME_G_ALPHA_I_SIGNALLING_EVENTS	1.97.E-03
REACTOME_BIOLOGICAL_OXIDATIONS	5.20.E-03
REACTOME_BASE_EXCISION_REPAIR	5.73.E-03
REACTOME_MITOCHONDRIAL_FATTY_ACID_BETA_OXIDATION	5.79.E-03

REACTOME_EARLY_PHASE_OF_HIV_LIFE_CYCLE	7.53.E-03
REACTOME_PTM_GAMMA_CARBOXYLATION_HYPUSINE_FORMATION_AND_ARYLSULFATASE_ACTIVATION	7.71.E-03
REACTOME_ADENYLATE_CYCLASE_INHIBITORY_PATHWAY	1.22.E-02
REACTOME_A_TETRASACCHARIDE_LINKER_SEQUENCE_IS_REQUIRED_FOR_GAG_SYNTHESIS	1.29.E-02
REACTOME_GLYCOPROTEIN_HORMONES	3.54.E-02
REACTOME_ERKS_ARE_INACTIVATED	3.57.E-02
REACTOME_ACTIVATION_OF_GENES_BY_ATF4	3.64.E-02
REACTOME_PLATELET_SENSITIZATION_BY_LDL	4.16.E-02
