# Supplementary material for: Classification and Functional Analysis between Cancer and Normal Tissues Using Explainable Pathway Deep Learning through RNA-Sequencing Gene Expression
Source: Int J Mol Sci. 2021 Oct 26;22(21):11531. doi: 10.3390/ijms222111531 (PMC8584109; doi:10.3390/ijms222111531)
Supplement: Supplementary file 1 [file ijms-22-11531-s001.zip › TableS4.pdf]

Table S4 Gene Expression Data

Project	Normal	Cancer	Total
GTEx	7,429	363	7,792
K-562	0	70	70
TARGET	11	723	734
TCGA	727	9,808	10,535
**Total**	**8,167**	**10,964**	**19,131**
