# Supplementary material for: Classification and Functional Analysis between Cancer and Normal Tissues Using Explainable Pathway Deep Learning through RNA-Sequencing Gene Expression
Source: Int J Mol Sci. 2021 Oct 26;22(21):11531. doi: 10.3390/ijms222111531 (PMC8584109; doi:10.3390/ijms222111531)
Supplement: Supplementary file 1 [file ijms-22-11531-s001.zip › TableS5.pdf]

Table S5 MSigDB with TCGA TARGET GTEx

Molecular signature collection	Number of Pathway	Number of Pathway Member genes	Number of pathway member genes common to TCGA TARGET GTEx genes
c2 reactome	674	6,025	5,796
c2 kegg	186	5,266	5,227
c4 cgn	427	4,880	4,756
c1 positional	326	30,010	22,197
c2 cgp	3,433	20,623	18,512
c2 cp	1,329	8,904	1,275
c2 cp biocarta	217	1,279	8,620
c3 mir	221	7,444	7,135
c3 tft	615	12,774	12,180
c4 cm	431	8,405	8,142
c5 GO bp	4,436	15,578	15,005
c5 GO cc	580	12,198	11,722
c5 GO mf	901	14,549	14,081
c6 oncogenic signatures	189	11,250	10,570
c7 immunologic signatures	4,872	20,652	18,141
